# Life Cycle Analysis on Fossil Energy Ratio of Algal Biodiesel: Effects of Nitrogen Deficiency and Oil Extraction Technology

**DOI:** 10.1155/2015/920968

**Published:** 2015-04-27

**Authors:** Hou Jian, Yang Jing, Zhang Peidong

**Affiliations:** ^1^South China Green Design R&D Center, China Quality Certification Center Guangzhou Branch, Guangzhou, Guangdong 510620, China; ^2^Qingdao Institute of Bioenergy and Bioprocess Technology, Chinese Academy of Sciences, Qingdao, Shandong 266101, China; ^3^Qingdao University of Science & Technology, Qingdao, Shandong 266042, China

## Abstract

Life cycle assessment (LCA) has been widely used to analyze various pathways of biofuel preparation from “cradle to grave.” Effects of nitrogen supply for algae cultivation and technology of algal oil extraction on life cycle fossil energy ratio of biodiesel are assessed in this study. Life cycle fossil energy ratio of *Chlorella vulgaris* based biodiesel is improved by growing algae under nitrogen-limited conditions, while the life cycle fossil energy ratio of biodiesel production from *Phaeodactylum tricornutum* grown with nitrogen deprivation decreases. Compared to extraction of oil from dried algae, extraction of lipid from wet algae with subcritical cosolvents achieves a 43.83% improvement in fossil energy ratio of algal biodiesel when oilcake drying is not considered. The outcome for sensitivity analysis indicates that the algal oil conversion rate and energy content of algae are found to have the greatest effects on the LCA results of algal biodiesel production, followed by utilization ratio of algal residue, energy demand for algae drying, capacity of water mixing, and productivity of algae.

## 1. Introduction

With the rapid growth of economy and energy consumption, petroleum resources are gradually depleted and environmental pollution is increasingly serious. It has become emergent to search for alternative energy especially in the field of traffic and to mitigate the environmental problems caused by fossil energy production and using. Biomass energy has the characteristics of renewable raw material and biological carbon sequestration. Development of bioenergy is considered as an effective way to solve energy shortage and improve environment.

Changes of land use and increased emission of greenhouse gases can be caused by production of transportation biofuels from terrestrial energy plants [1]. Using algae as a feedstock for biofuels has led to much excitement and initiative. Although algae based fuels are widely considered as clean energy, fossil energy input during production of biofuels from algae may still aggravate depletion of nonrenewable resources and pollution of environment [2]. It is very necessary to estimate the ratio of energy output to fossil energy consumption (fossil energy ratio) of algal biodiesel based on the concept of life cycle analysis.

Currently, several studies of life cycle analysis on algal biofuels have been carried out. Frank et al. [3], Yang et al. [4], and Clarens et al. [5, 6] have shown that fertilizer input contributes a lot to the overall life cycle fossil energy consumption and global warming of algal biofuel. Sander and Murthy [7] have shown that extraction of oil from dried algae results in high life cycle fossil energy input. With the increasing researches on mass culture of algae and algal oil extraction, several studies have assessed the life cycle fossil energy ratio of algal biofuels produced by new technologies. Life cycle assessment results of Lardon et al. [8] on algal biodiesel produced from* Chlorella vulgaris* with different nitrogen (N) supplies have shown that life cycle fossil energy ratio can be improved when algae grow under low N condition. Some researches show that the productivities, constituents, and calorific values of different algae species may present different changing tendency when the N supply reduces [9–11]. Therefore, in order to identify whether life cycle fossil energy ratio of algal biodiesel can be promoted by low N condition, energy analysis of biodiesel based on different algae species should be carried out. Batan et al. [12] and Brentner et al. [13] compared the life cycle fossil energy use of algal biodiesel by extraction of oil from dried and wet algae. However, the energy consumption for extraction of oil from wet algae is hypothetical data, and reliability of the assessment results remains unknown.

It is thus clear that the existing LCA studies on algal biofuels contain several problems and this results in the fact that life cycle fossil energy ratio of algal biofuel cannot be scientifically identified based on the present research status. To fill up the deficiency above, with algal biodiesel as the objective of our study, we investigate the cell compositions and productivities of* Phaeodactylum tricornutum* and* Chlorella vulgaris* grown with sufficient and limited nitrogen supply. Studies on lipid extraction from wet algae may mainly concern extraction yields of algal oil but often neglect energy required for lipid extraction [14]. In this study, energy demands for extraction of oil from wet algae of pilot production are monitored. Effects of nitrogen supply conditions and algal oil extraction technologies on life cycle fossil energy ratio of algal biodiesel are assessed. To verify the reliability of our study, our results are compared with energy balance of other similar LCA studies on algal biofuel. A sensitivity analysis is performed to identify key parameters affecting life cycle fossil energy ratio of algal biodiesel.

## 2. Methodologies

### 2.1. Functional Unit

The functional unit for the LCA in this study is 1 MJ biodiesel produced.

### 2.2. Life Cycle System Boundary of Algal Biodiesel

**Figure 1:**
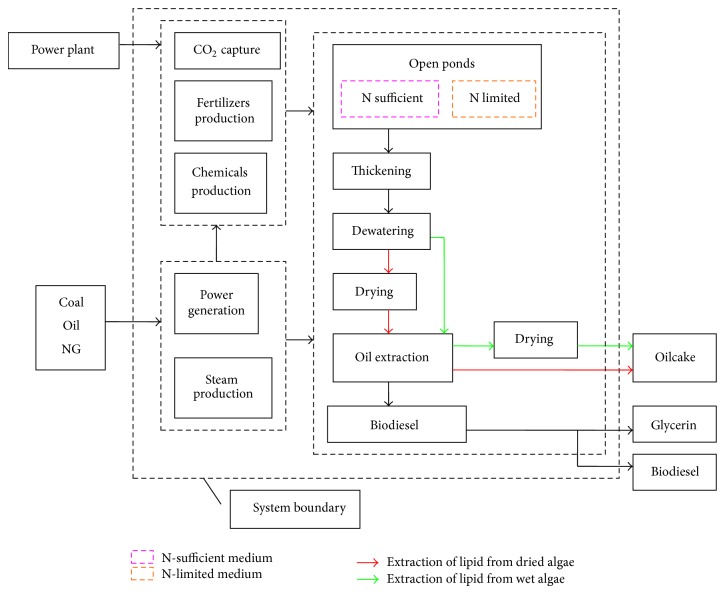
Life cycle system of biodiesel production from algae.

Figure 1 shows the simulated life cycle system of algal biodiesel production in this study. Algae are grown in open ponds with sufficient or limited nitrogen supply. 50% of the normal nitrogen supply is used in the N-limited medium. Algae harvesting includes steps of concentration, dewatering, and drying [15]. The content of algal biomass in the fluid from cultivation ponds is lower than 5 wt%. Concentration reduces the water content of the algal biomass from 99 wt% to 95 wt%. Dewatering is needed to further decrease the water content to 60 wt%–80 wt%. Algae have to be dried up to a 90 wt% solid content if the same technology as soybean lipid extraction is applied to extraction of oil from algae. Chen et al. [16] designed the extraction of oil from wet algae biomass with about 30 wt% solids. Biodiesel is obtained through transesterification reaction of algal lipid and methanol.

### 2.3. Evaluation Model for Life Cycle Energy Efficiency of Algal Biodiesel

#### 2.3.1. Life Cycle Primary Energy Consumption Calculation

When 1 MJ biodiesel is produced, the life cycle primary fossil energy demand (EC_LC_) is calculated as the sum of all the primary fossil energy consumptions due to production of all the process energy and materials directly used in all the substages according to the GREET model [17]:

(1) $$\begin{array}{c} \text{E}{\text{C}}_{\text{LC}}=\sum_{i}\sum_{j}{\text{EE}}_{i,j}\times {\text{PE}}_{j}+\sum_{i}\sum_{n}M_{i,n}\times {\text{PE}}_{n}, \end{array}$$

where EE_*i*,*j*_ is the process energy *j* consumption during substage *i* (MJ); PE_*j*_ is the life cycle primary fossil energy use for process energy *j* production (MJ/MJ); *M*_*i*,*n*_ is the material *n* consumption during substage *i* (kg); PE_*n*_ is the life cycle primary fossil energy use for material *n* production (kg/MJ).

During algae cultivation, the power demand for mixing (EE_mixing_) is computed using

(2) EEmixing=Malgae×Wmixing×tw×trC=Malgae×Wmixing×twYV,

where *M*_algae_ is the algae consumption to produce 1 MJ biodiesel (kg); *W*_mixing_ is the mixing capacity (W/m^3^); *t*_*w*_ is the working hours of mixing equipment per day (h/d); *t*_*r*_ is the retention time of algae (d); *C* is the algal biomass concentration (kg/m^3^); *Y*_*V*_ is the volumetric productivity (kg/m^3^·d).

The *M*_algae_ and *Y*_*V*_ are calculated using the following equations, respectively:

(3) Malgae=1HVbiodiesel×ηester×ηextra×ηharve×Palgae,oil×Pnuetr oil,

(4) $$\begin{array}{c} Y_{V}=Y_{A}\times \frac{A}{V}, \end{array}$$

where HV_biodiesel_ is the net caloric value of biodiesel (MJ/kg); *η*_harve_, *η*_extra_, and *η*_ester_ are the efficiencies of algae harvesting, algal oil extraction, and esterification, respectively (%); *P*_algae,oil_ is the total oil content of algae (%); *P*_nuetr oil_ is the percentage of neutral oil in total oil (%); *Y*_*A*_ is the areal productivity (kg/m^2^·d); *A*/*V* is the ratio of illuminated area to volume (m^−1^).

Power consumption for pumping (EE_pumping_) is calculated using

(5) EEpumping=Malgae×ρwater×g×HC×ηpump×1+λwater,evap+λlose,

where *ρ*_water_ is the density of water (kg/m^3^); *g* is the force of gravity (N/kg); *H* is the liquid head (m); *η*_pump_ is the pumping efficiency (%); *λ*_water,evap_ and *λ*_water,lose_ are water evaporation rate and water delivering loss rate, respectively (%).

When 1 MJ biodiesel is produced, consumptions of CO_2_ (*M*_CO_2__) and fertilizer (*M*_fertili_) are calculated by using the following equations, respectively:

(6) $$\begin{array}{c} M_{\text{C}{\text{O}}_{2}}=\frac{M_{\text{algae}}\times P_{\text{algae},\text{C}}\times 44/12}{\eta_{\text{fixing}}}, \end{array}$$

(7) $$\begin{array}{c} M_{\text{ferti}}=M_{\text{algae}}\times P_{\text{algae},\text{N}(P)}\times \left(\lambda_{\text{N}\left(P\right),\text{evap}}+\eta_{\text{harve}}\right), \end{array}$$

where *P*_algae,C_ is the carbon content of algae (%); *η*_fixing_ is the CO_2_ fixing efficiency of algae (%); *P*_algae,N(*P*)_ is the nitrogen or phosphorus content of algae (%); *λ*_N(*P*),evap_ is the nitrogen or phosphorus evaporation rate (%).

#### 2.3.2. Life Cycle Energy Output Calculation

The life cycle energy outputs are calculated based on the energy released from combustion of biodiesel, oilcake, and glycerin [8, 18]:

(8) $$\begin{array}{c} {\mathrm{EP}}_{\text{biodiesel}}=1, \end{array}$$

(9) $$\begin{array}{c} {\mathrm{EP}}_{\text{oilcake}}=M_{\text{algae}}\times {\text{HV}}_{\text{algae}}-\frac{1}{\eta_{\text{esterification}}}, \end{array}$$

(10) $$\begin{array}{c} {\mathrm{EP}}_{\text{glycerin}}=M_{\text{glycerin}}\times {\text{HV}}_{\text{glycerin}}, \end{array}$$

where *EP*⁡_biodiesel_, *EP*⁡_oilcake_, and *EP*⁡_glycerin_ are the energy released from biodiesel, oilcake, and glycerin combustion, respectively (MJ); HV_algae_ is the net caloric value of algae (MJ/kg); *M*_glycerin_ is the glycerin output when 1 MJ biodiesel is produced (kg); HV_glycerin_ is the net caloric value of glycerin (MJ/kg).

HV_algae_ is calculated as

(11) $$\begin{array}{c} \text{H}{\text{V}}_{\text{algae}}=\sum_{i}P_{\text{algae},n}\times {\text{HV}}_{n}, \end{array}$$

where *P*_algae,*n*_ is the percentage of ingredient *n* in algae (%); HV_*n*_ is the net caloric value of ingredient *n* (MJ/kg).

#### 2.3.3. Life Cycle Fossil Energy Ratio Calculation

The life cycle fossil energy ratio of biodiesel production (*η*_fossil_) is the ratio of the life cycle energy output to the life cycle primary fossil energy consumption:

(12) $$\begin{array}{c} \eta_{\text{fossil}}=\frac{\sum_{m}{\mathrm{EP}}_{m}}{\sum_{i}{\text{EC}}_{i}}\times 100\%, \end{array}$$

where *EP*⁡_*m*_ is the energy output *m* (MJ); EC_*i*_ is the primary energy consumption in substage *i* (MJ).

## 3. Data Collection

Under conditions with sufficient and limited nitrogen supply, the algal productivities and cell compositions of* Phaeodactylum tricornutum* and* Chlorella vulgaris* grown in open ponds are shown in Table 1. The chemical formulas and net caloric values of carbonhydrate, protein, and lipid are according to Lardon et al. [8]. Phosphorus content of algae is 1 wt% [19]. Algae concentration is 0.5 g/L and pond height is 0.2 m. According to Fagerstone et al. [20], when algae are cultivated in open ponds, the cumulative N_2_O emissions over the light and dark periods are 1.53 × 10^−5^ kg and 6.51 × 10^−6^ kg per kg N input, respectively. When the concentration of CO_2_ injected to algae cultivation ponds is 5%, CO_2_ fixing efficiencies of different algae species have been shown in Table 2.

According to the base data in Tables 1 and 2 and formulas of (6) and (9), the calculated nitrogen fertilizer inputs and heat values of algae under conditions of normal and limited N supply are shown in Table 3. As Tables 1 and 3 show, under low N condition, lipid content of* Chlorella vulgaris* increases and algae productivity drops; nitrogen fertilizer and heat value of* Phaeodactylum tricornutum* both decrease.

Operation capacity of paddle wheel and aeration in open ponds is 3.72 W/m^3^ [25]. It is assumed that working time of mixing equipment is 12 h per day. Average delivery head of centrifugal pump is 7.5 m with efficiency of 70% [15]. Water evaporation rate during algae cultivation is 10% [6] and water delivering loss is 5% [3].

Energy consumed in algae harvesting is from literature [26]. Dissolved air flotation is used for algae concentration with an electricity requirement of 100 kWh/t dry mass. The electricity demand of centrifuge for algae dewatering is 37 kWh/t dry mass. The energy demand for thermal drying of algae to 10% water content is 615.6 kWh/t dry mass. 10 wt% and 5 wt% of the algal cells are lost in concentration and dewatering, respectively [27].

Energy demands for extraction of oil from dried and wet algae are listed in Table 4. It is assumed that the percentage of neutral lipid in total lipid of algae is 80%. Energy requirements for oil refining are according to literature [28]. Energy consumptions in oil conversion stage are from literature [29]; the conversion efficiency is 96.5% and the net calorific value of biodiesel is 37.2 MJ/kg.

It is assumed that electricity and steam consumed in the assessed system are generated from coal in China. The fertilizers and chemicals are produced using technologies on world average level and the energy demands for fertilizers and chemicals production are from Gabi database [31]. CO_2_ applied to algae growth is assumed to be from flue gas discharged from power plant. Flue gas from power or steel plant generally contains substances like sulfur oxide, nitric oxide, and heavy metals which are deleterious to algae growth. CO_2_ needs to be separated from the flue gas before it is injected into algae cultivation ponds. Membrane separation of CO_2_ is used with steam demands of 73 kWh per ton of recovered CO_2_ and a capture efficiency of 85% [32]. CO_2_ capture not only provides nutrients for algae growth but also has been required in most coal-fired power stations. Energy demands for carbon capture are allocated between the power plant and the algae farm on an energy basis.

## 4. Results

### 4.1. Energy Efficiency Comparison Analysis of Biodiesel Production from Algae Grown with Normal and Limited Nitrogen Supply

**Figure 2:**
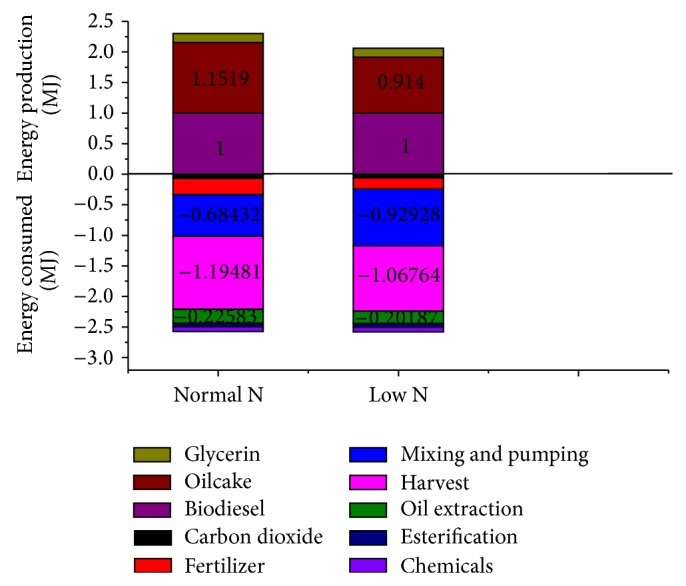
*Phaeodactylum tricornutum*. Energy losses and gains for the production of biodiesel from* Phaeodactylum tricornutum* grown in N-sufficient and N-limited mediums.

**Figure 3:**
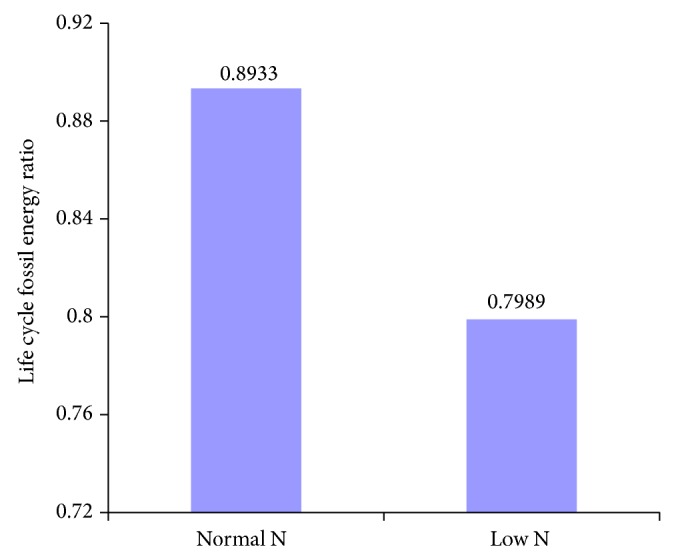
Life cycle fossil energy ratio for the production of biodiesel from* Phaeodactylum tricornutum* grown in normal and limited nitrogen supply conditions.

**Figure 4:**
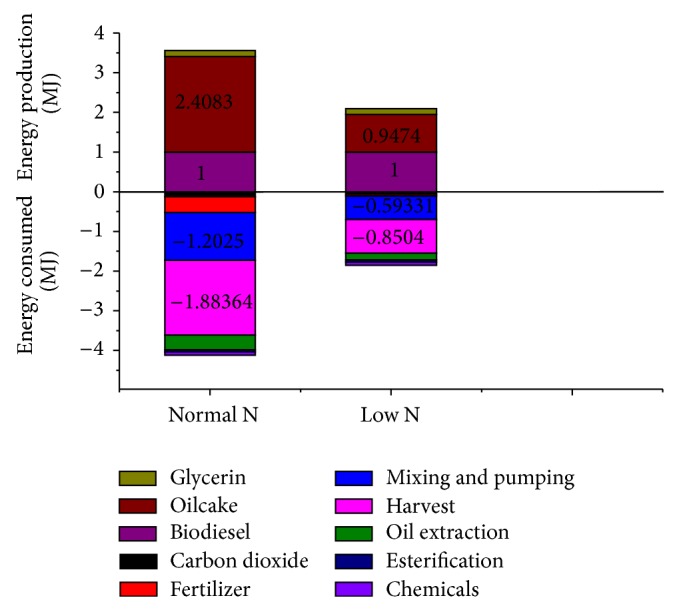
*Chlorella vulgaris*. Energy losses and gains for the production of biodiesel from* Chlorella vulgaris* grown in N-sufficient and N-limited mediums.

**Figure 5:**
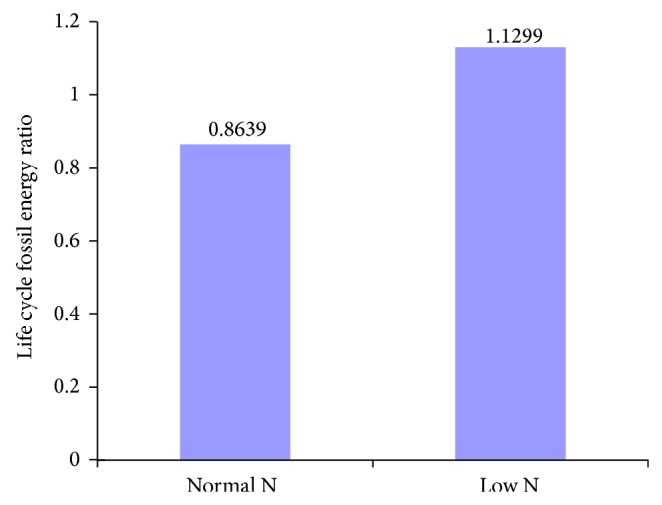
Life cycle fossil energy ratio for the production of biodiesel from* Chlorella vulgaris* grown in normal and limited nitrogen supply conditions.

With open ponds cultivation of algae, chemical absorption of CO_2_, and extraction of oil from dried algae, the calculated life cycle energy production and fossil energy consumed for* Phaeodactylum tricornutum* and* Chlorella vulgaris* based biodiesel with different nitrogen supplies are shown in Figures 2 and 4, and life cycle fossil energy ratios are shown in Figures 3 and 5.

It can be seen from Figure 2 that, under limited nitrogen supply condition, fossil energy consumption for harvesting and oil extraction of* Phaeodactylum tricornutum* and energy production of algae biomass all decrease. This is mainly due to the fact that oil content of* Phaeodactylum tricornutum* increases under low N condition and less algae input for 1 functional unit of biodiesel production is required. However, for the productivity and heat value of* Phaeodactylum tricornutum* both decrease under low N condition, the decline rate of energy production of algal biomass (11.06%) is higher than that of the energy required in algae harvesting and oil extraction (10.64%), and energy consumption for mixing of cultivation water increases by 35.8%. As a result, life cycle fossil energy ratio for* Phaeodactylum tricornutum* based biodiesel with limited nitrogen supply decreases by 10.56% compared with normal nitrogen supply (Figure 3).

As can be seen from Figure 4, under limited nitrogen supply condition, energy consumption for harvesting and oil extraction of* Chlorella vulgaris* decreases by 54.85% because of higher lipid content compared to normal nitrogen supply. Due to the heat value of* Chlorella vulgaris* increase under low N condition, the total energy production of biodiesel and oilcake only decreased by 14.09%. As a result, life cycle fossil energy ratio for* Chlorella vulgaris* based biodiesel under limited nitrogen supply increases by 30.78% compared with normal nitrogen supply (Figure 5).

### 4.2. Energy Efficiency Comparison Analysis of Biodiesel Production from Oil Extracted from Dried and Wet Algae

**Figure 6:**
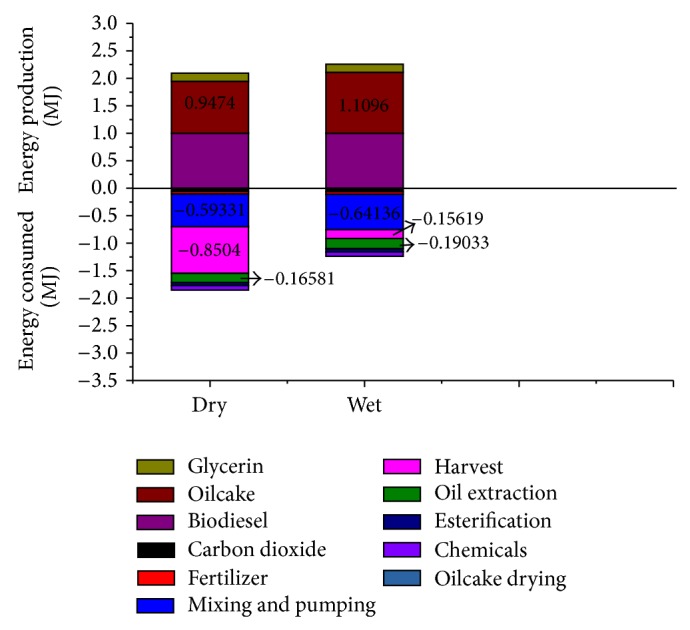
Energy input and output of biodiesel production using oil extracted from dried and wet algae.

**Figure 7:**
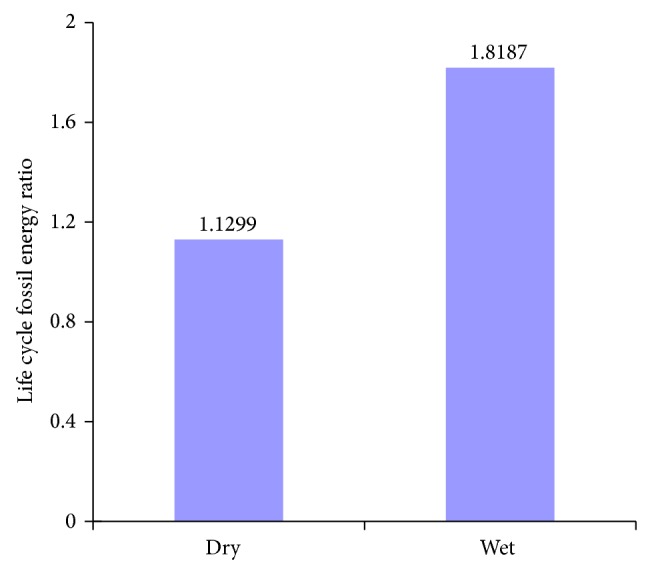
Life cycle fossil energy ratio for the production of biodiesel from algal oil extracted from dried and wet algae.

The calculated life cycle energy outputs and fossil energy consumed for algal biodiesel produced from* Chlorella vulgaris* under low N condition in open ponds, with CO_2_ from membrane separation, and oil extracted from dried and wet algae are shown in Figure 6, and life cycle fossil energy ratios are shown in Figure 7.

As can be seen from Figure 6, compared to extraction of oil from dried algae, the energy consumed for extraction of oil from wet algae with subcritical cosolvents increases by 14.79% compared to extraction of oil from dried algae, and energy required for mixing increases by 8.1%. This is mainly due to the fact that efficiency for oil extraction from wet algae is lower than from dried algae and more algae input for 1 functional unit of biodiesel production is required. However, algae drying process omitted makes lipid extraction from wet algae perform a 43.83% improvement in the life cycle fossil energy ratio of algal biodiesel compared to extraction of oil from dried algae (Figure 7).

### 4.3. Comparison of the Results with Other LCA Studies of Algal Biodiesel

This section has the goal of comparing the results of this study with other similar LCA studies on algal biodiesel and then analyzing the main differences among values for the life cycle fossil energy ratios in different studies. Values of energy demands, energy outputs, and life cycle fossil energy ratios of algae based biodiesel production from two literatures have been collected and are compared in Table 5.

As can be seen from Table 5, when algal biodiesel made from similar pathways is taken as the research object, life cycle fossil energy ratio of biodiesel produced from dried algae is 66.18% higher in this study than in Lardon et al.'s study, and life cycle fossil energy ratio of biodiesel produced from wet algae is 35.82%, 355%, and 28.57% higher in this study than in studies of Lardon et al., Razon and Tan [31], and Batan et al., respectively.

High energy consumption caused by backward algae drying technology is the main reason for the lower life cycle fossil energy ratio of biodiesel produced from dried algae in Lardon et al.'s study. Compared to energy consumption data of algae drying in the study of Zhao and Hu in 2009 on energy consumption of sludge treatment in wastewater treatment plant, energy consumption data of algae drying in study of Lardon et al. is from experimental study of Hassebrauck et al. in 1996 on sludge drying by belt dryer and its energy consumption for algae drying is about 2 times higher than results in study of Zhao and Hu [26]. So timeliness of basic data has important effects on the validity of LCA results of algal biodiesel. Compared to energy consumption data of extraction of oil from wet algae in this study based on pilot-scale study of Chen et al., Lardon et al. [8] and Batan et al. [12] calculated the energy consumption for extraction of oil from wet algae based on hypothesis and both of their results are higher than the energy consumed for extraction of oil from wet algae with subcritical cosolvents in study of Sturm and Lamer [15].

In study of Yang et al. [14], complicated algae cultivation process and low yield of algae lead to the high energy input during stages of algae cultivation and oil extraction. It makes algal biodiesel not able to deliver more energy than is required to produce it.

### 4.4. Sensitivity Analysis

**Figure 8:**
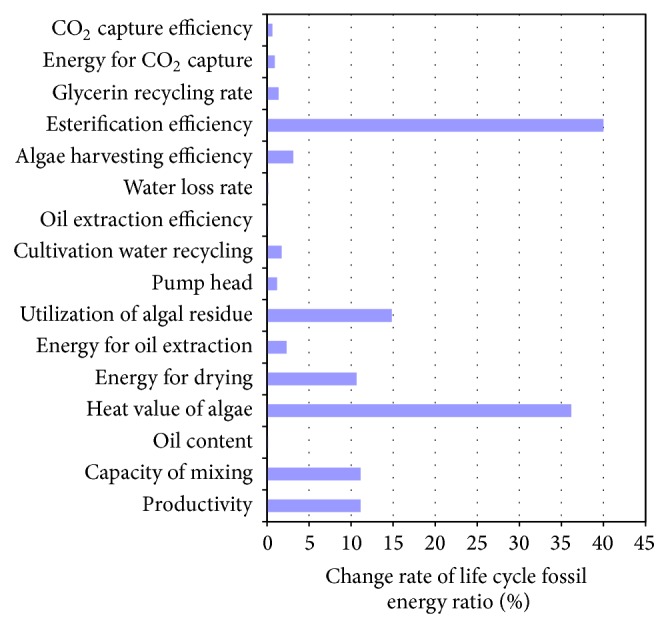
Sensitivity analysis of effects of several parameters on life cycle fossil energy ratio of algae biodiesel.

A sensitivity analysis is performed to determine key parameters affecting the life cycle fossil energy ratio of algal biodiesel (see Figure 8). All parameters analyzed vary over equal confidence intervals. The effects of different parameters will be ranked by the change in the life cycle fossil energy ratio of algal biodiesel. Algal biodiesel produced from* Chlorella vulgaris* under low N condition in open ponds, with CO_2_ from membrane separation, and oil extracted from dried algae have been taken as the baseline scenario. The change rate of all uncertain parameters is 40%.

As can be seen from Figure 8, the changes of esterification efficiency and heat value of algae are found to have the greatest effects on the life cycle fossil energy ratio of algal biodiesel. As esterification efficiency and heat value of algae decrease by 40%, the life cycle fossil energy ratio of algal biodiesel changes by 40% and 36.17%, respectively. The second important parameters are utilization ratio of algal residue, algae cultivation water recycling rate, energy demand for algae drying, capacity of mixing, and productivity of algae and when those parameters separately decrease by 40%, the life cycle fossil energy ratio of algal biodiesel changes between 10 and 15%. When algal oil content, oil extraction energy consumption, pump head, cultivation water recycling rate, oil extraction efficiency, cultivation water loss, algae harvesting efficiency, glycerin recycling rate, CO_2_ capture energy consumption, and CO_2_ capture efficiency separately decrease by 40%, the life cycle fossil energy ratio of algal biodiesel changes under 5%.

## 5. Conclusions

(1) Nitrogen deficiency can not only promote the oil content of many species of algae but also decrease the productivity of algae. The change of algae cell composition has certain effects on its energy output. Life cycle fossil energy ratio of biodiesel produced from* Chlorella vulgaris* grown under nitrogen-limited conditions increases by 30.78%. Life cycle fossil energy ratio of biodiesel produced from* Phaeodactylum tricornutum* grown with nitrogen deprivation decreases by 10.56%.

(2) Compared to extraction of oil from dried algae, extraction of oil directly from wet algae with subcritical cosolvents can effectively promote the life cycle fossil energy ratio of algal biodiesel.

(3) Comparison of the results with other LCA studies of algal biodiesel shows that, when algal biodiesel made from similar pathways is taken as the research object, life cycle fossil energy ratio of biodiesel produced from dried algae is 66.18% higher in this study than in Lardon et al.'s. Worse timeliness of data source for energy consumption of algae drying is the main reason. Life cycle fossil energy ratio of biodiesel produced from wet algae is 35.82% and 28.57% higher in this study than in the studies of Lardon et al. and Batan et al., respectively. Compared to Lardon et al. [8] and Batan et al. [12]'s calculation of the energy consumption for extraction of oil from wet algae based on hypothesis, energy consumption data of extraction of oil from wet algae in this study is based on pilot-scale study of Chen et al. So the results are more reliable.

(4) The changes of esterification efficiency and heat value of algae have the greatest effects on the life cycle fossil energy ratio of algal biodiesel, followed by utilization ratio of algal residue, algae cultivation water recycling rate, energy demand for algae drying, capacity of mixing, and productivity of algae. When esterification efficiency and heat value of algae decrease by 40%, the life cycle fossil energy ratio of algal biodiesel changes by 40% and 36.17%, respectively.

## Figures and Tables

**Table 1:** Productivity and cell composition of algae grown under N-sufficient and N-limited conditions.

Strains	Normal N supply				Limited N supply (50% of normal N supply)			
	Protein/%	Carbonhydrate/%	Lipid/%	Productivity/g/m^2^·d	Protein/%	Carbonhydrate/%	Lipid/%	Productivity/g/m^2^·d
*Phaeodactylum tricornutum* ^b^	32.2	18.5	28.46	25	24.76	16.7	31.8	15.45
*Chlorella vulgaris* ^c^	31	51	18	22	6	54	40	19.85

^b^Source: [9, 21]. ^c^Source: [11, 22].

**Table 2:** CO_2_ fixing efficiencies of different algae species.

	*Phaeodactylum tricornutum *	*Chlorella vulgaris *
CO_2_ fixing efficiency/%	64.9^f^	60^g^

^f^Source: [23]. ^g^Source: [24].

**Table 3:** Net calorific values of algae and N fertilizer inputs.

Species of algae	Heat values and nitrogen contents	Normal N	Low N
*Phaeodactylum tricornutum *	Heat value (MJ/kg)	18.3	18.19
	N content (kg/kg)	0.052	0.040

*Chlorella vulgaris *	Heat value (MJ/kg)	18.33	23.27
	N content (kg/kg)	0.050	0.010

**Table 4:** Energy consumptions and efficiency for algal oil extraction.

	Extraction from dried algae^i^	Extraction from wet algae^j^
Power (kWh/t algae)	25	26.46
Steam (MJ/t algae)	1170.8	1239.01
Efficiency (%)	97.5	90

^i^The energy demands for extraction of oil from dried algae are from SEPA (State Environmental Protection Administration of China) of China [30].

^j^The energy demands for extraction of oil wet algae are based on a pilot-scale operation of algal oil extraction plant in China.

**Table 5:** The primary energy demands, energy outputs, and life cycle fossil energy ratios for algal biodiesel production in different researches.

Oil extraction technology	This study		Lardon et al., 2009 [8]		Yang et al., 2014 [14]	Batan et al., 2010 [12]
	Dry	Wet	Dry	Wet	Wet	Wet
Basic condition						
Oil content/%	40		40		24	50
Productivity/g/m^2^·d	19.85		19.25		16	24.9
Primary fossil energy consumption/MJ						
Cultivation	0.65	0.71	0.41	0.59	2.246	0.73
Concentration	0.11	0.11	—	—	0.103	—
Dewatering	0.04	0.04	—	—	—	0.17
Drying	0.7	—	1.39	—	—	—
Oil extraction	0.17	0.19	0.14	0.52	1.895	0.21
Esterification	0.06	0.06	0.03	0.03	—	0.17
Fertilizer production	0.05	0.05	0.08	0.11	1.041	—
Chemicals production	0.08	0.08	0.27	0.43	0.443	—
Biogas generation	—	—	—	—	0.089	—
Sewage treatment	—	—	—	—	0.884	—
Energy production/MJ						
Biodiesel	1	1	1	1	1	1
Oilcake	0.9474	1.1096	0.57	1.23	—	0.79
Glycerin	0.1474	0.1474	—	—	0.565	—
Credit for “fresh” water	—	—	—	—	0.162	—
Biogas	—	—	—	—	1.378	—
Credit for ammonium compounds	—	—	—	—	0.027	—
Life cycle fossil energy ratio	1.13	1.82	0.68	1.34	0.4	1.4
